# Generation and Characterization of Bovine Testicular Organoids Derived from Primary Somatic Cell Populations

**DOI:** 10.3390/ani12172283

**Published:** 2022-09-03

**Authors:** Jahaira Cortez, Barbara Leiva, Cristian G. Torres, Víctor H. Parraguez, Mónica De los Reyes, Albert Carrasco, Oscar A. Peralta

**Affiliations:** 1 Department of Animal Production Sciences, Faculty of Veterinary and Animal Sciences, University of Chile, Santa Rosa 11735, Santiago 8820808, Chile; 2 Doctorate Program of Forestry, Agriculture, and Veterinary Sciences (DCSAV), University of Chile, Santa Rosa 11315, Santiago 8820808, Chile; 3 Department of Clinical Sciences, Faculty of Veterinary and Animal Sciences, University of Chile, Santa Rosa 11735, Santiago 8820808, Chile; 4 Department of Biological Sciences, Veterinary and Animal Sciences, University of Chile, Santa Rosa 11735, Santiago 8820808, Chile; 5 Laboratory of Animal Physiology and Endocrinology, Department of Animal Science, Faculty of Veterinary Sciences, Universidad de Concepción, Chillán 3780000, Chile

**Keywords:** bovine testicular organoid, 3D-culture system, Leydig cells, Sertoli cells, peritubular myoid cells

## Abstract

**Simple Summary:**

Organoids are 3D-cell culture systems composed of tissue-specific cells that create structures similar to those of their tissue of origin. Organoids have generated great interest in recent years, since they are considered a useful tool to perform laboratory studies in different scientific areas including reproduction. Testicular organoids (TOs) may provide an innovative model for the study of testicular physiology in various species; however, no previous studies have reported on the production of TOs in the bovine species. Thus, in the present study, we sought to generate and characterize bovine TOs, derived from testicular cell populations that include Leydig, Sertoli and peritubular myoid cells. After isolation from testes and characterization, testicular cells were cultured in ultra-low attachment dishes. Testicular cells formed TOs after 3 days of culture. Leydig, Sertoli and peritubular myoid cells displayed specific locations and changed in number in TOs. Moreover, bovine TOs were able to produce and increase the concentration of testosterone after 27 days of culture. The present study represents the first report on the generation and characterization of bovine TOs. These TOs could be useful tools to evaluate the impact of exogenous factors on the physiology of sperm production and testis development in domestic and wild cattle.

**Abstract:**

Organoids are 3D-culture systems composed of tissue-specific primary cells that self-organize and self-renew, creating structures similar to those of their tissue of origin. Testicular organoids (TOs) may recreate conditions of the testicular niche in domestic and wild cattle; however, no previous TO studies have been reported in the bovine species. Thus, in the present study, we sought to generate and characterize bovine TOs derived from primary testicular cell populations including Leydig, Sertoli and peritubular myoid cells. Testicular cells were isolated from bovine testes and cultured in ultra-low attachment (ULA) plates and Matrigel. TOs were cultured in media supplemented from day 3 with 100 ng/mL of BMP4 and 10 ng/mL of FGF2 and from day 7 with 15 ng/mL of GDNF. Testicular cells were able to generate TOs after 3 days of culture. The cells positive for STAR (Leydig) and COL1A (peritubular myoid) decreased (*p* < 0.05), whereas cells positive for WT1 (Sertoli) increased (*p* < 0.05) in TOs during a 28-day culture period. The levels of testosterone in media increased (*p* < 0.05) at day 28 of culture. Thus, testicular cells isolated from bovine testes were able to generate TOs under in vitro conditions. These bovine TOs have steroidogenic activity characterized by the production of testosterone.

## 1. Introduction

Organoids are 3D-culture systems composed of stem cells and/or tissue-specific primary cells that self-organize and self-renew, creating structures similar to those of their tissue of origin [1]. They bear either complete or partial structural and functional similarities with organs in vivo [2]. Organoids have generated great interest in recent years, since they are considered a useful tool to perform in vitro studies in different areas including toxicology, virology, oncology, microbiology and reproduction [3,4,5,6]. In particular, testicular organoids (TOs) have enabled the study of testicular development and have been used as a model for assaying the effects of different experimental factors and treatments [7,8,9]. In this regard, TOs may recreate conditions of the testicular niche, where spermatogenesis occurs through the self-renewing and differentiation of spermatogonial stem cells (SSCs) [10]. Furthermore, TOs may be a useful tool for analyzing strategies for preserving fertility and recovering sexual functionality [9,10]. This is especially important in the field of human reproductive and regenerative medicine, where TOs may allow the discovery of new treatments for infertility in men undergoing chemotherapy, azoospermia or trauma, among other injuries [10,11]. In the field of cattle reproduction, TOs may provide an innovative in vitro model for the study of spermatogenesis, factors associated with sperm production and eventually to develop new biotechnological applications for genetic improvement or wildlife conservation.

The testicular niche can be defined as the anatomical space where SSCs develop under the effect of signals originating from both the surrounding cells and the extracellular matrix [12,13]. In the case of testes, the SSC niche involves somatic cells including Leydig, Sertoli and peritubular myoid cells and structural components such as the basement membrane of the seminiferous tubule [14,15,16]. Sertoli cells are responsible for providing the necessary mechanical and nutritional support for SSC differentiation, being the only somatic cells in direct contact with SSCs [17]. Sertoli cells produce a variety of cytokines and growth factors, including bone morphogenetic protein 4 (BMP4), fibroblast growth factor 2 (FGF2) and glial cell line-derived neurotrophic factor (GDNF), that promote the proliferation and differentiation of SSCs [18,19]. Peritubular myoid cells are separated from Sertoli cells by the basement membrane, but together they make up the seminiferous tubules and produce components of the basement membrane [14,18,20]. In addition, peritubular myoid cell contraction promotes the expulsion of sperm during spermiation [21,22]. Leydig cells are located in the interstitial space of the seminiferous tubules, and like Sertoli and peritubular myoid cells, they produce several growth factors, including inhibin, insulin-like growth factors 1 (IGF1), insulin-like peptide 3 (INSL3), interleukin 1α (IL1α) and transforming growth factor β (TGFβ), that regulate SSC function [18]. In addition, Leydig cells are the primary source of androgens, which are necessary for the formation of sperm and reproductive function [23,24,25,26]. Nevertheless, SSC renewal and differentiation are complex and dynamic processes, but TOs may constitute a valuable system to recreate the testicular niche [26,27,28]. The organoid culture system could help to understand the interactions of germ cells with neighboring somatic cells and to analyze the effects of external and internal signals that control cell survival, differentiation and tissue development [29,30].

Despite the exponential increase in the number of reports on organoid derivation for in vitro studies, limited information has been published on TOs. To date, TOs have been mainly reported in humans, rodents and some domestic animal species including pigs and monkeys [31,32,33,34]. Nevertheless, to the best of our knowledge, currently there are no reports of TOs in the bovine species. The development of bovine TOs is not only relevant for understanding how internal and external factors affect testicular function in this species, but also as an alternative experimental model to study reproductive physiology in domestic and wild ruminants [35,36,37,38]. Moreover, due to the ability to mimic the structure of the testicular niche, bovine TOs may be used as a 3D culture system for in vitro SSC production, germ cell or gamete derivation. However, the particularities in the isolation, culture and long-term preservation of bovine testicular cells and the subsequent TO formation require exhaustive analysis. Thus, in the present study, we sought to generate and characterize bovine TOs derived from primary somatic cell populations including Leydig, Sertoli and peritubular myoid cells.

## 2. Materials and Methods

### 2.1. Ethics

All experimental procedures have been approved and were performed in accordance with the guidelines and regulations of the Bioethical Committees of the Faculty of Veterinary and Animal Sciences at the University of Chile.

### 2.2. Study Design

Testicular cells were isolated from bovine testes (*n* = 5) and cultured at a concentration of 1 × 10^6^ cells/well in Ultra Low Attachment (ULA) plates to form testicular organoids. Organoids were cultured (3 replicates) in DMEM F12 supplemented with 10% FBS, 100 IU/mL penicillin, 100 μg/mL streptomycin and 100 μg/mL amphotericin B. The culture medium from day 3 was supplemented with 100 ng/mL of BMP4 and 10 ng/mL of FGF2 and from day 7 with 15 ng/mL of GDNF. The testicular cell populations (Leydig, Sertoli and peritubular myoid cells) at day 0 were analyzed by Q-PCR for gene expression of STAR, WT1 and αSMA, and by flow cytometry and immunofluorescence for immunodetection of STAR, WT1 and COL1A. TOs were collected at days 3, 7, 14 and 28 of culture and examined by phase contrast microscopy for morphological characteristics, by Q-PCR for gene expression (10–12 TOs for each day) of STAR, WT1 and αSMA, and by confocal immunofluorescence (10–12 TOs for each day) for immunodetection of STAR, WT1 and COL1A.

### 2.3. Bovine Testis Collection

Testes were obtained by orchiectomy from healthy Angus bulls aging 7 to 10 months. Once collected, testes were washed and transported to the laboratory at 4 °C immersed in ice cold Hanks balanced salt solution free of calcium and magnesium (HBSS; Hyclone Laboratories, UT, USA) containing 100 μg/mL of streptomycin, 100 U/mL of penicillin and 100 μg/mL of amphotericin B (HBSS/P-E).

### 2.4. Isolation of Bovine Leydig, Sertoli and Peritubular Myoid Cells

The isolation of Leydig, Sertoli and peritubular myoid cells was performed according to a previously reported sequential protocol with some modifications [39]. Briefly, the tunica albuginea of each testis was removed and a piece of parenchyma (12–15 grams) was excised and transferred to a beaker. Then, the parenchyma was vigorously sectioned until small pieces of tissue were obtained, which were resuspended in 100 mL of ice cold HBSS/P-E. The sectioned tissue was allowed to settle twice by gravity for 15 min and the supernatant was removed. Subsequently, 300 mL of HBSS/P-E was added and the suspension was placed in orbital agitation at 37 °C for 20 min. The supernatant was removed, and the pellet was subjected to enzymatic digestion using 80 mL of HBSS/P-E, 10 mL of Type IV Collagenase (1%), 10 mL of 2.5% Trypsin-EDTA and 2 mL of DNAse (1 mg/mL) under stirring for 15 min at 37 °C. The tissue was then passed through a metal strainer into a new beaker and an equivalent volume of DMEM-F12 supplemented with fetal bovine serum (FBS) was added to stop enzymatic digestion. The obtained solution was centrifuged at 200× *g* for 5 min and the supernatant was removed. The obtained pellet was resuspended in 200 mL of DMEM-F12 supplemented with 10% FBS, 100 μg/mL of streptomycin and 100 U/mL of penicillin and allowed to settle for 30 min. The supernatant was collected and the cells were allowed to settle for 20 min to later obtain Leydig cells. Then, the pellet obtained was resuspended in 100 mL of PBS containing 1 M glycine and 2 mM EDTA (pH 7.4) for 10 min and then allowed to settle for 15 min. This step was repeated twice, and the supernatant obtained was centrifuged at 200× *g* for 5 min to obtain peritubular myoid cells. These cells were seeded in minimum essential medium (MEM) supplemented with 10% FBS, 100 μg/mL of streptomycin, 100 U/mL of penicillin and 100 μg/mL of amphotericin B. The pellet obtained, corresponding to Sertoli cells, was resuspended in 10 mL of DMEM-F12 supplemented with 100 μg/mL of streptomycin, 100 U/mL of penicillin and 100 μg/mL of amphotericin B. Leydig cells obtained from the supernatant by sedimentation and subsequent centrifugation for 5 min at 200× *g* were then purified by centrifugation with discontinuous percoll gradients (100, 60, 34, 26 and 21%) in PBS for 30 min at 1500× *g*. The cells obtained between the 26 and 34% and 34 and 60% fractions were collected, centrifuged at 200× *g* for 10 min and then cultured in DMEM-F12 supplemented with 10% FBS, 100 μg/mL of streptomycin and 100 U/mL of penicillin. The cell culture media was removed, and fresh media was added every 2–3 days.

### 2.5. Three-Dimensional Co-Culture of Testicular Cells

Testicular primary Leydig, Sertoli and peritubular myoid cells obtained by enzymatic digestion and maintained for up to 5–6 passages were used to generate TOs. Leydig, Sertoli and peritubular myoid cells (3 × 10^6^ cells for each type) were resuspended together and brought to a concentration of 1 × 10^6^ cells/mL in culture medium containing DMEM-F12 supplemented with 10% FBS, 100 IU/mL penicillin, 100 μg/mL streptomycin and 100 μg/mL amphotericin B. Then, 100 μL of the cell suspension was placed in each well of a 96-well ULA U-shape button plate (Corning, NY, USA) for culture. On day 7 of culture, cell aggregates in each well were transferred to 40 μL of Matrigel growth factor reduced (Corning, NY, USA) and incubated for 10–20 min at 36 °C until solidification. Once solidified, the TOs were transferred to 6-cm ULA plates. Cultures were maintained at 36 °C under a humidified atmosphere with 5% CO_2_ for another 21 days.

### 2.6. Testicular Organoid Culture Conditions

DMEM-F12 supplemented with 10% FBS, 100 IU/mL penicillin, 100 μg/mL streptomycin and 100 μg/mL amphotericin B was used as the basic culture medium. The medium was changed every 2 or 3 days. The basic culture medium was supplemented from day 3 of culture with 100 ng/mL of BMP4 and 10 ng/mL of FGF2 (R&D Systems, Minneapolis, MN, USA) (refer as: BF medium) and from day 7 of culture with 15 ng/mL of GDNF (R&D Systems) (refer as: BFG medium).

### 2.7. Testicular Organoid Whole-Mounting Staining

To examine the structure of TOs and the distribution of testicular cell types (Leydig, Sertoli and peritubular myoid cells), these structures were fixed overnight in 4% paraformaldehyde at 4 °C for 1 h. After fixation, antigen retrieval was performed in 10 mM sodium citrate aqueous solution with 0.05% Tween-20 (P/N K38485272825, Merck, Frankfurt, Germany) with pH 6 for 15 min at 95 °C. The samples were cooled down for 15–30 min and then washed 3 times with PBS and 0.1% Tween-20. Permeabilization was performed using Triton X-100 (P/N 9036-19-5, Merck) in 1X PBS for 2 h at RT. Blocking was performed by incubation with 2% BSA in PBS for 1 h at RT. Proteins of interest were immunodetected using primary antibodies diluted in 2% BSA (Table 1) for 3 days at 4 °C. Samples were then washed 3 times with 0.1% Tween in PBS. Later, secondary antibodies diluted in 2% BSA were incubated for 2 days at 4 °C. Samples were counter-stained using Pure Blu DAPI Nuclear Staining Dye (P/N 135-1303, BioRad, Hercules, CA, USA). 

### 2.8. Digital Image Acquisition and Processing

Digital images were acquired using a contrast phase microscope connected to a digital camera (Motic, Xiamen, China). To assess organoid size, their area was determined with ImageJ software (National Institute of Health, Bethesda, MD, USA, v1.47). Digital 3D images of TOs were obtained using a Zeiss LSM 700 confocal microscope (Carl Zeiss, Oberkochen, Germany). The proportions of cells expressing STAR, WT1 and COL1A were determined by dividing the number of marker-positive cells by the number of cells stained with DAPI on three randomly selected sections.

### 2.9. Q-PCR Analysis

Cell samples and TOs were fixed in lysis buffer (Thermo Fisher Scientific, Waltham, MA, USA) with 20 µL of β-Mercaptoethanol (Sigma-Aldrich, St. Louis, MO, USA). The RNA from the cells was isolated using the GeneJet RNA purification kit (Thermo Fisher). The total mRNA was quantified using a Qubit 3.0 Fluorometer (Thermo Fisher). For the removal of genomic DNA, a DNAse I kit (Thermo Fisher) was used. The cDNA was synthesized and amplified using an Affinity Script Q-PCR cDNA Synthesis Kit (Agilent Technologies, Santa Clara, CA, USA), with 1000 ng of RNA as input and using a TC1000-G thermocycler (DLab, PEK, Beijing, China). Samples were analyzed for the gene expression of β-ACTIN (housekeeping), and cell-specific markers STAR (Leydig cells), WT1 (Sertoli cells) and α-SMa (peritubular myoid cells) using Q-PCR (Table 2). The PCR reaction was performed using a Brilliant SYBR Green QPCR Master Mix kit (Agilent Technologies). Each RT-PCR reaction (10 μL) contained the following: 2X Brilliant II SYBR Green QPCR master mix (5 μL), target forward primer (200 nM), target reverse primer (200 nM), cDNA synthesis reaction (1 μL) and nuclease-free PCR grade water to adjust the final volume. The PCR amplification was carried out in an Eco Real-Time PCR System (Illumina Incorporated, San Diego, CA, USA). The thermal cycling conditions were 95 °C for 10 min, followed by 40 repetitive cycles at 95 °C for 30 s, and 60 °C for 1 min. In each experiment, the level of gene expression was recorded as CT values that corresponded to the number of cycles where the fluorescence signal can be detected above a threshold value. The relative quantification of the target gene expression across treatments was evaluated using the comparative ΔΔCT method. The CT value was determined by subtracting the β-ACTIN CT value (most stable endogenous gene CT value) from the target CT value of the sample. The calculation of ΔΔCT involved using the target gene expression of untreated testicular cells as an arbitrary constant to subtract from all other CT sample values.

### 2.10. Fluorescence Activated Cell Sorting (FACS) Analysis

The quantification of the populations of Leydig, Sertoli and peritubular myoid cells was performed by the determination of the proportions of cells positive for the expression of STAR, WT1 and COL1A markers, respectively. The cells were detached after incubation with Trypsin for 10–20 min at 38 °C. The cells were then fixed with 4% paraformaldehyde in PBS for 15 min at 4°C, washed with PBS and stored at 4 °C overnight. Later, cells were permeabilized in PBS with 1% Triton X-100 for 15 min at room temperature after fixing and incubated with primary antibodies (Table 1) overnight at 4 °C. The cells were then stained in the dark with a fluorescent secondary antibody for 1 h at room temperature. After incubation, the cells were washed with PBS and centrifuged to remove unbound antibody. The cells were resuspended in 1 mL of PBS and analyzed in a FACSCalibur (BD Biosciences, Erembodegem, Belgium) using FlowJo v10 (Tree Star, Ashland, OR, USA) software.

### 2.11. Testosterone Production Assay

Testosterone production in the TO culture medium was measured at 8, 11, 14, 18, 21 and 27 days of culture using enhanced chemiluminescence technology (Vitros Immunodiagnostic Product Testosterone kit, Ortho-Clinical Diagnostics, Bridgend, UK) following manufacturer instructions. The bound horseradish peroxidase (HRP) conjugate was determined by a luminescence reaction using luminol reactive. An electron transfer reagent was present to enhance the level of light produced prolonging its emission spectra. The amount of HRP conjugate bound is in direct proportion to the concentration of testosterone present in the sample and was analyzed with VITROS Immunodiagnostics testosterone calibrators.

### 2.12. Statistical Analysis

Statistical analyses were performed on relative gene expression, TO diameter and percentage of cells positive for testicular cell-specific markers using InfoStat software (Infostat, Version 2018; Córdoba, Argentina). The means values for each replicate were analyzed by one-way ANOVA. Gene expression values between days of culture, TO diameter and percentage of positive cells were analyzed using Tukey’s multiple comparison test (*p* < 0.05).

## 3. Results

### 3.1. Isolation and Characterization of Bovine Leydig, Sertoli and Peritubular Myoid Cells

**Figure 1:**
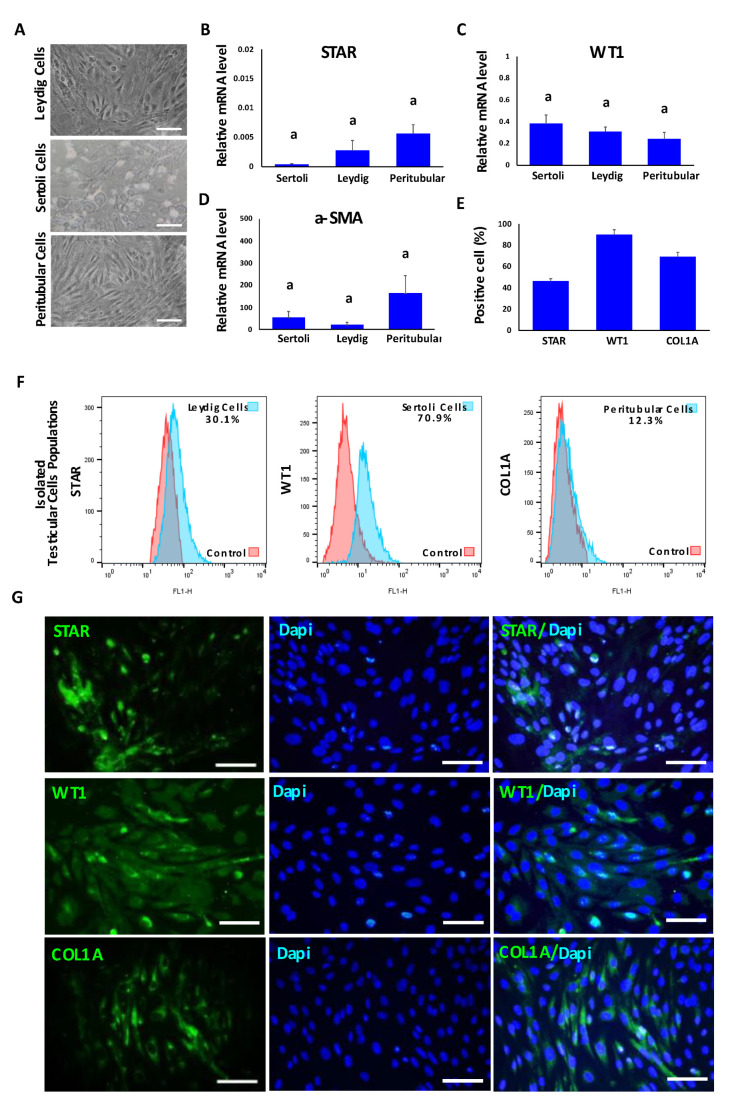
Characterization of primary bovine testicular cell types including Leydig, Sertoli and peritubular myoid cells used for TO formation. (**A**) Phase contrast representative images of Leydig, Sertoli and peritubular myoid primary cell cultures. (**B**–**D**) Gene expression analysis of STAR (Leydig cells), WT1 (Sertoli cells) and α-SMA (peritubular myoid cells) detected no significant differences among cell cultures. Data were expressed as the mean ± SEM (*n* = 3). (**E**) Percentages of STAR, WT1 and COL1A immune-positive cells in respective Leydig, Sertoli and peritubular myoid cell cultures. (**F**) Representative histograms of testicular cell populations positive for each marker: STAR, WT1 and COL1A (Leydig, Sertoli and peritubular cells, respectively). (**G**) Representative immunofluorescence analysis of STAR, WT1 and COL1A markers in Leydig, Sertoli and peritubular myoid cells. Superscript (a) indicates no significant (*p* > 0.05) differences in gene expression among cell types. Scale bar = 100 μm.

In order to determine the purity of the testicular cell populations used for TO formation, primary cell cultures were analyzed by using phase contrast microscopy, Q-PCR, immunofluorescence and FACS analyses. All testicular cells were plastic-adherent and while Leydig and peritubular myoids cells showed an epithelioid and slightly elongated form, Sertoli cells displayed a more rounded or cubical shape (Figure 1A). Gene expression levels of STAR, WT1 and α-SMA were detected in all cell cultures and no significant differences were detected among cell types (Figure 1B–D). The respective proportions of cells positive to STAR, WT1 and COL1A1 were analyzed in Leydig, Sertoli and peritubular myoid cell cultures on the total number of DAPI-stained nuclei for each culture (Figure 1E,G). The results revealed that 45.8% ± 5 of cells expressed STAR, 89.5% ± 2.6 of cells expressed WT1 and 69.1% ± 4.4 of cells expressed COL1A in Leydig, Sertoli and peritubular myoid cell cultures, respectively. Testicular cells were also characterized by FACS and 30.1% ± 4.5 of Leydig cell cultures were positive for STAR, 70.9% ± 3.4 of Sertoli cell cultures were positive for WT1 and 12.3% ± 2.2 of peritubular myoid cell cultures were positive for COL1 (Figure 1F).

### 3.2. Formation and Morphological Characterization of Multicellular Bovine TOs

**Figure 2:**
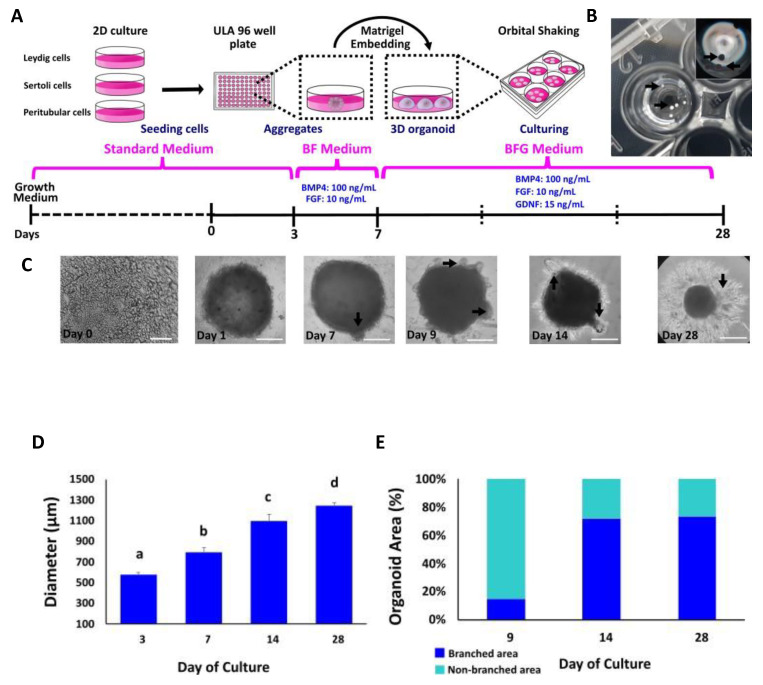
Generation and morphological evaluation of bovine TOs derived from primary bovine testicular cells. (**A**) Schematic diagram depicting the main steps for bovine TO production. (**B**) Spherical TO after 3 days of culture in ULA plates. (**C**) Representative bright-field images of morphological changes and differences in cellular reorganization in TOs. Arrows indicate branch formation from days 7 to 28 after treatment with FGF2, BMP4 and GDNF. (**D**) Average diameter of TOs at different days of culture. (**E**) Percentage of branched and non-branched area on TOs at 9, 14 and 28 days of culture. Bright field at day 0 (Scale bar = 100 μm). Data were expressed as the mean ± SEM (*n* = 3). Days 1, 7, 9 and 28 (Scale bar = 500 μm). Different superscripts (a, b, c and d) indicate significant (*p* < 0.05) differences in diameter among days of culture.

In order to establish a method to reproducibly generate uniform bovine TOs, we followed previously reported protocols for producing TOs with modifications [30,31,32,33]. Organoids were generated from primary cell cultures, harvested after 3, 7, 14 and 28 days of culture and examined for morphological characteristics and internal organization (Figure 2A). TOs appeared uniform and tightly packed, were spherical in shape and formed external branches (Figure 2B,C). The diameter used for TO size determination included both branched and unbranched areas. The mean diameter of TOs were 578 μm ± 12.7 at day 3, 794 μm ± 27.6 at day 7, 1096 μm ± 38.3 at day 14 and 1245 μm ± 14.2 at day 28 (Figure 2D). Once the organoids were transferred to Matrigel growth factor reduced and were treated with FGF2, BMP4 and GDNF, they presented a branched structure (black arrows in Figure 2C). By day 9 and 14, more branches were visible within the structure and by day 28, TOs increased the size of the branches, most probably due to extracellular matrix (ECM) production (Figure 2C). The proportion of branched in comparation to non-branched area increased during culture from 15% (Day 9) to 72% (Day 14) and 73% (Day 28). 

### 3.3. Testicular Cell Marker Evaluation by QPCR in Bovine TOs

**Figure 3:**
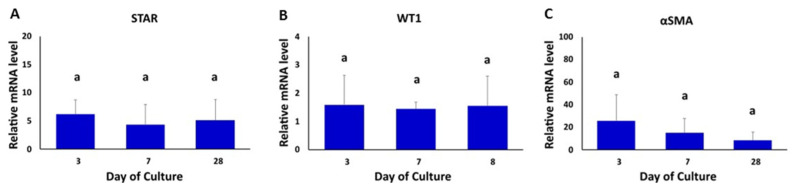
Gene expression analysis of testicular cell-specific markers in bovine TOs. (**A**–**C**) The gene expression levels of STAR (Leydig cells), WT1 (Sertoli cells) and α-SMA (peritubular myoid cells) were not different (*p* > 0.05) in TOs at days 3, 7 and 28 of the culture. β-ACTIN was used as a housekeeping gene. Gene expression values were normalized (fold-change) with respect to values in testicular cells isolated prior to the formation of TOs (control). Data were expressed as the mean ± SEM (*n* = 3). Superscript (a) indicates no significant (*p* > 0.05) differences in gene expression among days of culture.

The gene expression of Leydig, Sertoli and peritubular myoid cell markers (STAR, WT1 and α-SMA, respectively) were also evaluated in TOs at 3, 7 and 28 days of the culture (Figure 3). The levels of mRNA of STAR, WT1 and α-SMA were detected in all days of organoid culture and were not significantly different among days of culture (Figure 3A–C).

### 3.4. Confocal Immunofluorescence Analysis of Cell-Specific Testicular Markers in Bovine TOs

**Figure 4:**
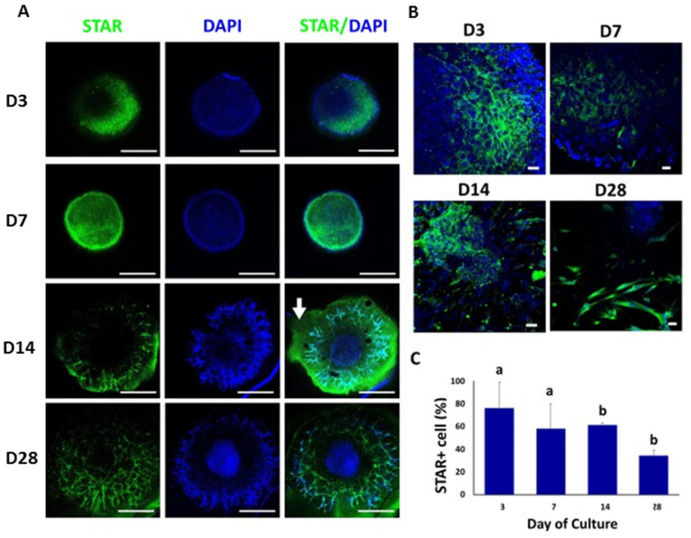
Confocal immunofluorescence analysis of STAR expression in whole mounted bovine TOs. (**A**,**B**) Immunofluorescence associated with STAR in whole TOs (**A**) and branched areas of TOs (**B**). (**C**) The percentages of STAR-positive cells on days 14 and 28 were significantly lower compared to days 3 and 7. Data were expressed as the mean ± SEM (*n* = 3). Cell nuclei were stained blue with DAPI. Scale bars = 500 μm (**A**) and 25 μm (**B**). White arrow = Matrigel area. Superscripts (a,b) indicate significant (*p* < 0.05) differences in proportions of cells positive for STAR between days of culture.

On day 3 of culture, we observed a patch of STAR-positive cells on a section of the peripheral region of the TOs (Figure 4A,B). From day 7, the STAR-positive cells migrated to the peripheral and partially to the central region of the organoid. Thereafter, these cells were found almost exclusively in the peripheral areas and branches from day 14 to day 28 of culture. The percentages of STAR-positive cells in TOs were 76% ± 22.9 (day 3), 58% ± 21.8 (day 7), 61% ± 2.0 (day 14) and 34% ± 4.8 (day 28) (Figure 4C). The percentages of STAR-positive cells on days 14 and 28 were lower (*p* < 0.05) compared to days 3 and 7 (Figure 4C).

**Figure 5:**
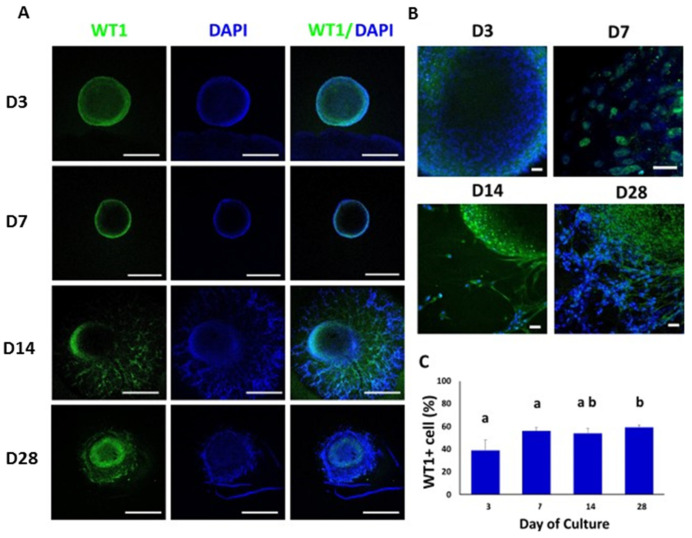
Confocal immunofluorescence analysis of WT1 expression in whole mounted bovine TOs. (**A**,**B**) Immunofluorescence associated with WT1 in whole TOs (**A**) and specific areas (**B**). (**C**) The percentages of WT1-positive cells on days 14 and 28 were significantly higher compared to days 3 and 7. Data were expressed as the mean ± SEM (*n* = 3). Cell nuclei were stained blue with DAPI. Scale bars = 500 μm (**A**) and 25 μm (**B**). Superscripts (a,b) indicate significant (*p* < 0.05) differences in proportions of cells positive for WT1 between days of culture.

Clumps of WT1-positive cells were observed mainly in the peripheral area of the TOs at 3, 7, 14 and 28 days of culture (Figure 5A,B). WT1 was also immunodetected in the branched area of TOs at days 14 and 28 of culture. The percentage of cells positive for WT1 were 39% ± 9.4, 56% ± 2.6, 54% ± 3.5, and 59% ± 1.8 (Days 3, 7, 14 and 28, respectively) (Figure 5C). The percentage of WT1-positive cells increased (*p* < 0.05) from days 3 and 7 to 28.

**Figure 6:**
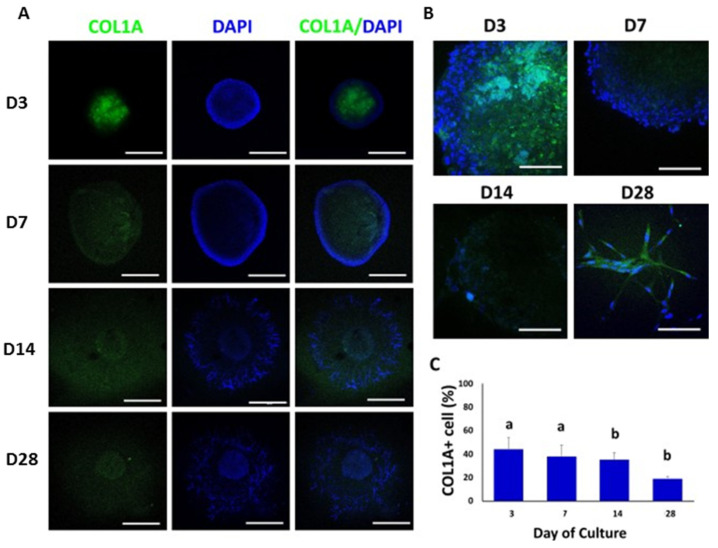
Confocal immunofluorescence analysis of COL1A expression in whole mounted bovine TOs. (**A**,**B**) Immunofluorescence associated with COL1A was observed in whole TOs (**A**) and specific areas (**B**). (**C**) The percentage of COL1A-positive cells decreased (*p* < 0.05) on days 14 and 28 compared to days 3 and 7. Data were expressed as the mean ± SEM (*n* = 3). Cell nuclei were stained blue with DAPI. Scale bars = 500 μm (**A**) and 25 μm (**B**). Superscripts (a,b) indicate significant (*p* < 0.05) differences in proportions of cells positive for COL1A between days of culture.

COL1A was immunolocalized at day 3 of culture in the central region of the TOs. COL1A-positive cells were decreasing during culture and cells immunoreactive to COL1A were not observed at day 28 of culture (Figure 6A,B). The percentages of COL1A-positive cells were 44% ± 9.9, 38% ± 9.8, 35% ± 5.9, and 19% ± 1.6 at days 3, 7, 14 and 28, respectively (Figure 6C). The percentage of COL1A-positive cells decreased (*p* < 0.05) from days 3 and 7 to days 14 and 28.

### 3.5. Testosterone Measurements in Testicular Organoids

**Figure 7:**
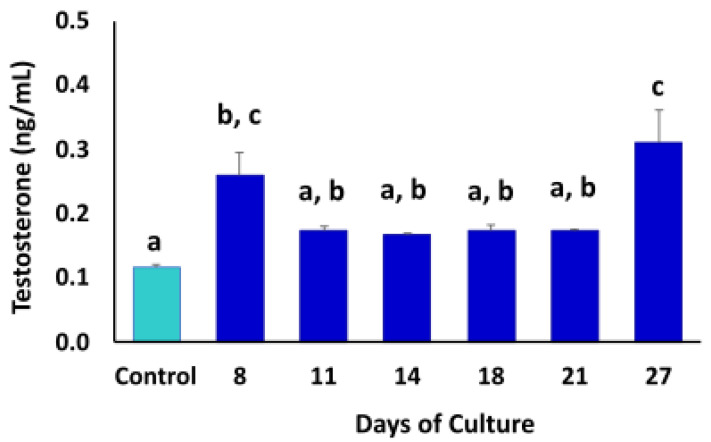
Chemiluminescence analysis of testosterone concentrations in culture medium of TOs during culture period. Higher (*p* < 0.05) levels of testosterone were detected at days 8 and 27 of culture. Fresh culture medium was used as control. Data were expressed as the mean ± SEM (*n* = 3). Superscripts (a–c) indicate significant (*p* < 0.05) differences in testosterone concentrations in TO culture media between days of culture.

The steroidogenic activity in the TOs was quantified by measuring testosterone concentrations in the culture media throughout the culture period (Figure 7). Fresh culture medium was used as control. Testosterone levels were higher (*p* < 0.05) at days 8 (0.26 ± 0.061) and 27 (0.31 ± 0.09) compared to the other days of culture and control.

## 4. Discussion

Organoids allow the replication of physiological and pathological processes under in vitro conditions through the recreation of cell-to-cell and matrix-to-cells interactions normally found in tissues and organs [40,41,42]. Cell organization and processes, however, may differ according to the methodology used for cell isolation and organoid culture [43,44,45,46,47]. In the present study, bovine Leydig, Sertoli and peritubular myoid cells were isolated from testes and cultured separately, mixed at a concentration of 1 × 10^6^ cells/mL, cultured for 7 days and then transferred to Matrigel for further 3D organization. This protocol differs from those commonly used in 3D testicular models, where testicular cells are obtained through enzymatic digestion and the remotion of connective tissue [6,8,30,32,34,48]. These cell isolation protocols may unintentionally provide other cell types to the testicular cell mix, including endothelial-like or uncharacterized cell types that may interfere with further experiments [8,30,31,32,49]. Following our testicular cell isolation method, we obtained cultures with populations of 21–37% of Leydig cells, 65–77% of Sertoli cells and 10–16% of peritubular myoid cells. These values are similar for Leydig and Sertoli cells to those obtained in a previous study that used a similar cell isolation protocol [39]. However, the proportions of peritubular myoid cells obtained here were lower (10–17%) compared to those previously reported (30–60%) [39]. The differences between these studies may be associated with the methodology used for cell detection, since COL1A immunodetection by FACS used in our study may be more specific for the identification of peritubular myoid cells compared to alkaline phosphatase staining and visual counting used in the previous study.

Previous studies focused on the generation of TOs have reported the resuspension of testicular cells directly in Matrigel or agarose [6,31,50,51,52]. In these studies, the time reported for TO formation varies between 2 and 9 days depending on the substrate or culture method and the number of cells used [30,31,32,33,40]. In comparison, our bovine TOs were initially produced by culturing dissociated cells on ULA well plates, where they aggregated at the center of the well, maximizing cell-to-cell contact and facilitating the formation of 3D cell aggregates. Accordingly, the time of bovine TO formation using this protocol was only 24 h, indicating that increasing cell proximity may result in improved efficiency for the production of TOs. A similar time period for TO formation could be obtained using the hanging drop method; however, these TOs also display highly variable sizes and shapes [30,32,33,53,54].

Cell density is one of the most critical aspects for organoid formation. Low cell density may result in failure to generate the organoid and in comparison, high density may predispose the formation of a necrotic core as a consequence of the lack of nutrient or oxygen diffusion to the center of the organoid [54,55,56,57]. In TOs, high cell density is important because it allows the tubulogenic formation; thus, cell number is a critical aspect for successful TO formation and must be chosen correctly [8,31,55]. The cell densities used in previous studies ranged from 1 × 10^3^ to 44 × 10^6^ and were effective for the formation of TOs [8,10,30,31,32,33,58]. We used 1 × 10^5^ cells per organoid, which allowed high efficiency for TO production and avoided the presence of necrotic cores in the formed TOs.

The use of Matrigel during culture increases the area where cells can reorganize and permits the formation of larger TOs. Despite the fact that the comparison of diameters and areas of TOs among studies is difficult due to the lack of measurements reported, previous studies have indicated diameters between 100 and 600 μm and average areas around 2966–4628 μm^2^ [7,33,34,50,59]. In our study, TOs presented diameters that ranged between 555.15 μm and 1272.88 μm. Diameters similar to ours have been observed in brain organoids; however, these structures have been designed to display larger diameters to better recreate the complexity of the brain tissue [60,61,62,63]. The increased diameters observed in our TOs could be attributed to the high proliferative or migratory activity of testicular cells; however, further analyses are required to elucidate these properties in bovine TOs [64].

Due to the multicellular nature of the 3D organoid cultures, we evaluated cell-specific gene and protein expression using testicular cell-specific markers. We compared changes in gene expression at days 3, 7 and 28 of culture to determine the location and fate of Leydig, Sertoli and peritubular myoid cells in the bovine TOs. The Q-PCR analysis revealed that mRNA levels of STAR, WT1 and α-SMA remained unchanged during the culture period. The relative expression of these genes could indicate that the 3D structure was able to maintain viable bovine testicular cells of each cell type during the entire culture period. STAR (steroidogenic acute regulatory protein) marker expression in bovine TOs suggests the presence of steroidogenic capacity of Leydig cells because this molecule is critically involved in testosterone biosynthesis [65,66]. STAR participates in the transfer of cholesterol from the outer to the inner membrane of mitochondria in Leydig cells [65]. Furthermore, the continuous expression of STAR and the increased concentration of testosterone in the culture medium indicates that Leydig cells present in bovine TOs display steroidogenic activity during TO formation and culture. Testosterone production in Leydig cells, cultured in vitro without LH stimulation, can vary between 0.4–0.8 ng/mL in rats, 0.68–5.7 ng/mL in humans and 0.7–0.11 ng/mL in cattle [33,48,67]. According to several studies, testosterone production in TOs, without LH stimulation, can vary between 0.07–1.5 ng/mL in humans, 2–5 ng/mL in pigs and 8.65–10.09 ng/mL in mice [6,8,33,50]. In our study, the concentration of testosterone in TO culture media ranged from 0.17 to 0.31 ng/mL, values that are similar to those previously reported. These results indicate that bovine TOs are able to exert steroidogenic activity, which is a fundamental property of the testis, especially for spermatogenic function.

One of the main objectives in the production of organoids is to generate structures that are biomimetic, so cell-specific location is crucial for the recreation of a functional testicular tissue. Confocal immunolocalization of testicular somatic cells in the bovine TOs revealed that STAR^+^ Leydig cells located in the periphery of TOs during the 28 days of culture. In previous studies, Leydig cells in TOs have been detected in the periphery and center as well as being randomly distributed within the organoid structure [30,32,54,55]. The specific cell location may be associated with the interaction with other cell types. In vivo Leydig cells are located in the interstitial space outside of the seminiferous tubules and they lack close interaction with Sertoli or peritubular myoid cells. Sertoli cells positive for WT1^+^ were localized in the periphery of TOs until day 28 of the culture, when cells were located in the central area. Despite the use of WT1 for Sertoli cell detection having not been previously reported in TOs, the location of Sertoli cells in our TOs is consistent with other studies [6,8]. Tubulogenic ability is defined as the capacity of the cells to self-assemble into testis cord-like structures and is considered as one of the three principal criteria to evaluate TO formation [8,33,49,68]. The degree of maturity of Sertoli cells is directly related to organoid assembly and tubulogenic ability [67,68]. In our study, we used Sertoli cells from bulls aging 7 to 10 months that are considered adult cells, so this could influence the testicular cytoarchitecture detected in bovine TOs and may determine the differences with other studies [8,31,42,48,55,68].

In the present study, COL1A^+^ peritubular myoid cells were located in the central area at day 3 and randomly distributed with lower intensity from days 7 to 28 of culture. The main marker used for the detection of peritubular myoid cells is α-SMA; however, COL1A is usually used as a complement for the detection of these cells [8,30,31,32,33,34,40,58,59,69]. However, some reports have used only COL1A as a marker for the detection of peritubular myoid cells in TOs [70]. Sertoli cells and peritubular myoid cells release components of the basement membrane; therefore, the deposition of ECM proteins is indicative of the function of these cells. In this regard, the protein expression of COL1A and gene expression of α-SMA mRNA could be indicators of the activity of peritubular myoid and Sertoli cells in the initial formation of the basal membrane; however, further characterization of basal membrane formation may be required [6,48,70].

Despite testicular cells in the bovine TOs being unable to reorganize into the typical testicular architecture, the generated TOs displayed clearly delineated central and exterior areas and showed branched-like peripheral structure. According to the different studies reported, the formation of a testicular architecture in TOs is highly variable. It has been reported that TOs formed from neonatal or prepubertal cells display more desirable outcomes than pubertal/adult testicular cells [30,31,32,47,48]. The reason for this difference is not entirely clear; however, it is believed that immature Sertoli cells may have higher morphogenic potential and may induce a stronger tubulogenic effect in TOs compared to other testicular cell types that have more passive or complementary roles [8,42,49,68].

## 5. Conclusions

Testicular cells isolated from bovine testes were able to generate TOs under in vitro conditions that included culture in ULA dishes and further disposition in Matrigel. Leydig, Sertoli and peritubular myoid cells displayed specific locations and changed in number in TOs. These bovine TOs have steroidogenic activity characterized by the production of testosterone into the culture media. The present method for TO generation could be used to track the location and fate of various cell populations and to examine the impact of exogenous factors in the physiology of testis formation.

## Figures and Tables

**Table 1:** Antibodies used for testicular cell-specific marker detection.

Antibody	Cell Marker	Cat.#	P/N Company	Dilution
Anti-COL1	Peritubular	SC293182	Santa Cruz Biotechonology	1:50
Anti-STAR	Leydig	AB96637	Abcam	1:100
Anti-WT1	Sertoli	AB89901	Abcam	1:100
IgG FITC	-	AB97050	Abcam	1:100
IgG Alexa Fluor 488	-	A32766	Thermo Fisher	1:500

**Table 2:** Sequence of primers used for Q-PCR analysis.

Gene	Nucleotide Sequence (5′-3′)	Accession Number
β-ACTIN	Forward CGCACCACTGGCATTGTCAT Reverse TCCAAGGCGACGTAGCAGAG	NM_173979.3
WT1 *	Forward AACCACACCACACCCATCC Reverse ACGCCGCACATCCTGAAT	XM_015466595.1
STAR *	Forward GACACGGTCATCACTCACGA Reverse TACGCTCACAAAGTCTCGGG	NM_174189.3
αSMA **	Forward CAGCCGAGAACTTTCAGGGAC Reverse GGTGATGATGCCGTGCTCTA	NM_001034502.1

* Wilms tumor. ** Steroidogenic acute regulatory protein.

## Data Availability

The data presented in this study are available on request from the corresponding author.
